# Supplementary material for: A Likelihood Approach for Real-Time Calibration of Stochastic Compartmental Epidemic Models
Source: PLoS Comput Biol. 2017 Jan 17;13(1):e1005257. doi: 10.1371/journal.pcbi.1005257 (PMC5240920; doi:10.1371/journal.pcbi.1005257)
Supplement: S1 File — (TAR.GZ) [file pcbi.1005257.s014.tar.gz › HSPH_Online-SI-Revision/Additions/MSS11/n100-extreme-SEITR_noisy/n100-extreme-SEITR_noisy_NEW2_table-8.pdf]

1	newly 3 weeks spec	2107.88	602.62	{2., 1160.}	72.0014	1.02114	newly 3 weeks cumul	3792.89	1178.77	{12., 2221.}	69.6637	0.898429	newly 5 weeks spec	7087.2	1546.48	{3., 2955.}	78.1792	1.19747	newly 5 weeks cumul	15 036.4	3714.33	{16., 7028.}	75.4563	1.05175	newly 58 weeks cumul	94 514.	67 408.6	{11 983., 87 198.}	28.6787	0.452211
2	newly 3 weeks spec	265.	223.7	{0., 288.}	145.721	1.53643	newly 3 weeks cumul	343.	318.7	{1., 437.}	149.469	1.31395	newly 5 weeks spec	3034.	1032.56	{0., 2224.}	104.679	2.10929	newly 5 weeks cumul	4334.	1905.	{2., 3484.}	112.677	1.83876	newly 66 weeks cumul	99 017.	63 051.6	{2., 92 580.}	36.3225	1.03617
3	newly 3 weeks spec	257.	2577.92	{0., 348.}	1066.56	1.93742	newly 3 weeks cumul	379.	3883.53	{0., 517.}	1087.5	1.72081	newly 5 weeks spec	2684.	1465.63	{0., 2801.}	122.798	2.4433	newly 5 weeks cumul	3894.	7561.76	{0., 5932.}	259.303	2.19411	newly 68 weeks cumul	98 993.	52 133.7	{0., 93 403.}	47.336	1.66603
4	newly 3 weeks spec	698.	351.41	{0., 374.}	123.082	1.86671	newly 3 weeks cumul	1030.	469.36	{2., 558.}	117.324	1.68248	newly 5 weeks spec	3933.	1522.3	{0., 2399.}	109.251	2.19955	newly 5 weeks cumul	6733.	2903.88	{2., 3940.}	114.133	1.95148	newly 66 weeks cumul	96 862.	59 893.5	{3., 91 923.}	38.1661	1.08623
5	newly 3 weeks spec	187.	2476.18	{0., 880.}	1372.36	1.73316	newly 3 weeks cumul	237.	3862.41	{1., 1100.}	1673.37	1.44923	newly 5 weeks spec	3231.	2174.61	{0., 7251.}	130.782	2.29374	newly 5 weeks cumul	4188.	8637.88	{1., 34 073.}	264.072	2.0141	newly 68 weeks cumul	98 880.	60 257.1	{1., 93 193.}	39.0603	1.13288
6	newly 3 weeks spec	323.	930.45	{0., 215.}	353.415	1.74408	newly 3 weeks cumul	460.	1635.81	{1., 305.}	419.063	1.52361	newly 5 weeks spec	3690.	1250.7	{0., 2163.}	106.122	2.30039	newly 5 weeks cumul	5281.	3918.24	{2., 3160.}	144.665	1.98942	newly 67 weeks cumul	95 041.	55 731.4	{2., 93 499.}	41.3607	1.32091
7	newly 3 weeks spec	2336.	1310.03	{67., 2799.}	52.4756	0.585708	newly 3 weeks cumul	4235.	2413.41	{133., 5142.}	51.6045	0.561147	newly 5 weeks spec	6848.	3501.61	{134., 7274.}	52.0086	0.661402	newly 5 weeks cumul	15 415.	8147.84	{354., 17 088.}	52.2928	0.609646	newly 59 weeks cumul	97 367.	75 547.6	{36 484., 92 738.}	22.4095	0.241424
8	newly 3 weeks spec	476.	133.23	{0., 265.}	97.5651	1.72869	newly 3 weeks cumul	697.	195.18	{3., 417.}	95.8537	1.49403	newly 5 weeks spec	4729.	812.26	{0., 1912.}	94.3916	2.28789	newly 5 weeks cumul	7052.	1376.54	{3., 2953.}	95.1254	1.96385	newly 66 weeks cumul	99 432.	65 187.2	{4., 91 803.}	34.4404	0.884558
9	newly 3 weeks spec	88.	1728.01	{0., 517.}	2005.47	1.56982	newly 3 weeks cumul	112.	2362.34	{1., 676.}	2145.41	1.25454	newly 5 weeks spec	2227.	2210.75	{0., 3703.}	161.37	2.36651	newly 5 weeks cumul	2748.	6565.45	{1., 8865.}	296.679	1.97565	newly 68 weeks cumul	99 354.	55 722.2	{1., 93 402.}	43.9155	1.44602
10	newly 3 weeks spec	982.	308.68	{0., 818.}	98.4623	1.90975	newly 3 weeks cumul	1566.	479.53	{2., 1251.}	96.2803	1.6948	newly 5 weeks spec	4646.	1288.17	{0., 4346.}	95.2064	2.26801	newly 5 weeks cumul	8317.	2470.62	{4., 7515.}	96.7306	1.97714	newly 64 weeks cumul	99 174.	54 844.9	{4., 90 034.}	44.6983	1.15767
11	newly 3 weeks spec	578.	105.26	{0., 186.}	94.8131	1.88175	newly 3 weeks cumul	848.	178.93	{2., 326.}	93.8809	1.53593	newly 5 weeks spec	4035.	402.59	{0., 657.}	93.8659	2.49461	newly 5 weeks cumul	6425.	793.02	{2., 1347.}	93.7619	2.00961	newly 63 weeks cumul	99 600.	52 595.5	{2., 88 723.}	47.1933	1.27569
12	newly 3 weeks spec	936.	161.17	{0., 209.}	97.7788	1.95342	newly 3 weeks cumul	1372.	258.14	{1., 347.}	96.9985	1.72665	newly 5 weeks spec	5911.	642.89	{0., 894.}	95.4229	2.44486	newly 5 weeks cumul	9781.	1254.13	{2., 1750.}	96.0569	2.1274	newly 64 weeks cumul	98 924.	56 065.3	{2., 88 835.}	43.3249	1.07662
13	newly 3 weeks spec	632.	391.97	{0., 511.}	127.568	1.71752	newly 3 weeks cumul	864.	570.1	{2., 840.}	128.218	1.48821	newly 5 weeks spec	4789.	1312.12	{0., 2587.}	98.8649	2.11505	newly 5 weeks cumul	7468.	2707.93	{3., 4658.}	106.029	1.89059	newly 65 weeks cumul	99 507.	63 091.8	{3., 92 351.}	36.5956	0.962497
14	newly 3 weeks spec	1910.	911.52	{58., 1970.}	55.4241	0.64324	newly 3 weeks cumul	3449.	1755.31	{144., 3791.}	53.5416	0.561726	newly 5 weeks spec	5421.	2375.7	{143., 4925.}	57.1219	0.719045	newly 5 weeks cumul	12 064.	5642.14	{375., 12 089.}	55.4717	0.623233	newly 58 weeks cumul	96 176.	74 471.9	{50 780., 89 833.}	22.5671	0.272074
15	newly 3 weeks spec	1491.	131.54	{0., 417.}	91.1777	1.91308	newly 3 weeks cumul	2425.	259.52	{2., 784.}	89.2981	1.73067	newly 5 weeks spec	6031.	354.37	{0., 1117.}	94.1242	2.33089	newly 5 weeks cumul	11 560.	830.45	{2., 2581.}	92.8162	2.04588	newly 59 weeks cumul	97 278.	48 562.8	{3., 85 051.}	50.0783	1.22101
16	newly 3 weeks spec	1486.	287.38	{0., 809.}	86.5976	1.7708	newly 3 weeks cumul	2628.	495.48	{1., 1421.}	86.0913	1.60741	newly 5 weeks spec	4920.	1004.22	{0., 3442.}	86.4707	1.88878	newly 5 weeks cumul	10 361.	2065.54	{3., 6602.}	86.1629	1.73863	newly 62 weeks cumul	92 728.	59 186.3	{6., 92 475.}	36.303	0.917675
17	newly 3 weeks spec	187.	2654.9	{0., 501.}	1476.47	1.81439	newly 3 weeks cumul	247.	3980.79	{1., 708.}	1665.53	1.533	newly 5 weeks spec	2188.	1453.03	{0., 1931.}	134.049	2.36644	newly 5 weeks cumul	3077.	7180.34	{1., 8794.}	297.069	2.05397	newly 68 weeks cumul	93 253.	51 371.4	{1., 92 195.}	44.9639	1.65842
18	newly 3 weeks spec	162.	421.76	{0., 259.}	316.42	1.52445	newly 3 weeks cumul	222.	528.03	{1., 376.}	289.257	1.31006	newly 5 weeks spec	2807.	2012.91	{0., 3328.}	142.063	2.30184	newly 5 weeks cumul	3654.	3800.65	{1., 4687.}	172.091	1.95967	newly 67 weeks cumul	99 640.	57 684.4	{2., 91 679.}	42.1072	1.34622
19	newly 3 weeks spec	726.	104.54	{0., 216.}	91.1129	1.76037	newly 3 weeks cumul	1060.	185.87	{2., 385.}	89.1066	1.45966	newly 5 weeks spec	4054.	364.84	{0., 809.}	93.5881	2.24892	newly 5 weeks cumul	6766.	748.24	{3., 1625.}	92.6267	1.85633	newly 62 weeks cumul	99 318.	54 695.8	{5., 88 955.}	44.9286	1.07572
20	newly 3 weeks spec	300.	591.34	{0., 210.}	259.893	1.81077	newly 3 weeks cumul	402.	783.92	{1., 301.}	253.91	1.48497	newly 5 weeks spec	3692.	1693.79	{0., 1670.}	116.816	2.44535	newly 5 weeks cumul	5182.	3616.59	{1., 2662.}	139.104	2.06489	newly 67 weeks cumul	98 887.	53 875.2	{2., 93 097.}	45.5184	1.50379
21	newly 3 weeks spec	120.	1720.36	{0., 220.}	1493.78	1.8803	newly 3 weeks cumul	157.	2802.11	{0., 274.}	1840.72	1.54204	newly 5 weeks spec	2417.	905.27	{0., 1018.}	113.137	2.63689	newly 5 weeks cumul	3205.	5189.76	{0., 2232.}	233.588	2.28177	newly 68 weeks cumul	99 034.	45 440.	{0., 90 287.}	54.1168	1.96836
22	newly 3 weeks spec	1137.	236.04	{0., 521.}	96.1935	1.8878	newly 3 weeks cumul	1829.	365.86	{1., 797.}	94.8004	1.77141	newly 5 weeks spec	5958.	1022.66	{0., 2925.}	94.0148	2.24156	newly 5 weeks cumul	10 630.	1934.37	{2., 5070.}	94.7194	2.04787	newly 64 weeks cumul	96 878.	58 097.7	{3., 91 335.}	40.03	0.95592
23	newly 3 weeks spec	1235.	351.07	{0., 621.}	99.8291	2.15811	newly 3 weeks cumul	1958.	503.04	{1., 948.}	97.3963	1.89018	newly 5 weeks spec	6598.	1591.58	{0., 4402.}	94.8924	2.50986	newly 5 weeks cumul	11 490.	2959.2	{2., 7028.}	96.6862	2.19284	newly 65 weeks cumul	88 617.	54 136.3	{2., 91 958.}	40.4528	1.24736
24	newly 3 weeks spec	102.	112.36	{0., 272.}	152.863	1.46208	newly 3 weeks cumul	138.	154.07	{1., 302.}	148.616	1.16836	newly 5 weeks spec	2027.	968.54	{0., 2735.}	111.12	2.31985	newly 5 weeks cumul	2665.	1483.55	{1., 3783.}	116.442	1.89337	newly 67 weeks cumul	98 866.	47 512.3	{1., 91 189.}	51.9428	1.72719
25	newly 3 weeks spec	917.	100.26	{0., 229.}	89.5594	1.85562	newly 3 weeks cumul	1410.	174.51	{2., 401.}	88.3709	1.624	newly 5 weeks spec	3941.	413.4	{0., 1118.}	90.7145	2.19241	newly 5 weeks cumul	7263.	798.05	{2., 1937.}	90.1163	1.91908	newly 63 weeks cumul	94 839.	55 167.1	{2., 89 466.}	41.8308	1.23949
26	newly 3 weeks spec	2138.	1527.48	{12., 3592.}	63.4864	0.737471	newly 3 weeks cumul	3825.	2626.26	{26., 6128.}	61.9205	0.700614	newly 5 weeks spec	6408.	4668.39	{18., 10 489.}	58.1284	0.758012	newly 5 weeks cumul	14 163.	10 151.1	{43., 23 275.}	60.203	0.721511	newly 61 weeks cumul	87 857.	75 728.3	{33 493., 92 666.}	16.2257	0.261229
27	newly 3 weeks spec	1999.	508.98	{0., 1548.}	81.0675	1.51942	newly 3 weeks cumul	3536.	894.14	{3., 2567.}	80.685	1.3533	newly 5 weeks spec	6511.	1631.04	{0., 5201.}	79.7822	1.65864	newly 5 weeks cumul	13 890.	3475.76	{4., 10 910.}	80.2477	1.47976	newly 61 weeks cumul	98 347.	64 016.	{6., 92 808.}	34.908	0.753594
28	newly 3 weeks spec	310.	3099.41	{0., 1176.}	1061.54	2.02251	newly 3 weeks cumul	474.	4710.95	{1., 1577.}	1054.66	1.77709	newly 5 weeks spec	2971.	1437.56	{0., 3726.}	117.235	2.60564	newly 5 weeks cumul	4384.	8559.48	{1., 17 329.}	259.062	2.2222	newly 68 weeks cumul	89 828.	48 016.9	{1., 91 930.}	47.3835	1.74835
29	newly 3 weeks spec	609.	426.24	{0., 984.}	126.91	1.71539	newly 3 weeks cumul	951.	612.24	{2., 1447.}	120.503	1.51091	newly 5 weeks spec	3642.	1858.74	{0., 7253.}	110.606	2.10035	newly 5 weeks cumul	6162.	3508.55	{3., 11 687.}	115.765	1.80981	newly 65 weeks cumul	99 052.	63 956.1	{3., 92 188.}	35.4318	0.922326
30	newly 3 weeks spec	109.	1682.93	{0., 399.}	1587.97	1.60062	newly 3 weeks cumul	138.	2699.99	{1., 528.}	1995.14	1.3411	newly 5 weeks spec	1649.	1928.71	{0., 4319.}	173.984	2.15095	newly 5 weeks cumul	2255.	6782.9	{1., 7306.}	354.065	1.89669	newly 68 weeks cumul	99 316.	55 995.9	{1., 93 407.}	43.6184	1.56738
31	newly 3 weeks spec	3137.	4492.04	{3375., 5197.}	47.4543	0.168223	newly 3 weeks cumul	6051.	8577.66	{6410., 9809.}	45.1598	0.160137	newly 5 weeks spec	6713.	8895.79	{7867., 10 003.}	39.2848	0.15012	newly 5 weeks cumul	17 548.	24 273.3	{19 706., 27 092.}	43.4764	0.158969	newly 58 weeks cumul	87 424.	82 666.4	{75 685., 92 340.}	11.4358	0.061212
32	newly 3 weeks spec	1418.	236.67	{0., 342.}	95.8244	2.05664	newly 3 weeks cumul	2225.	367.55	{2., 565.}	94.653	1.88406	newly 5 weeks spec	5244.	991.19	{0., 1861.}	95.3427	2.31116	newly 5 weeks cumul	10 289.	1897.28	{2., 3224.}	95.6427	2.10888	newly 64 weeks cumul	98 867.	56 979.8	{3., 91 535.}	42.3672	1.04946
33	newly 3 weeks spec	501.	785.15	{0., 94.}	240.054	2.34794	newly 3 weeks cumul	673.	995.12	{1., 150.}	229.807	1.99396	newly 5 weeks spec	5187.	700.36	{0., 1001.}	97.1205	2.97145	newly 5 weeks cumul	7427.	2421.12	{1., 1382.}	116.011	2.56435	newly 68 weeks cumul	99 003.	48 330.4	{1., 91 842.}	51.1829	1.94878
34	newly 3 weeks spec	1440.	289.94	{0., 693.}	95.6528	2.04939	newly 3 weeks cumul	2363.	445.75	{1., 1135.}	94.1485	1.90484	newly 5 weeks spec	6918.	1227.73	{0., 3702.}	93.7602	2.34699	newly 5 weeks cumul	12 627.	2341.96	{3., 6496.}	94.409	2.17782	newly 64 weeks cumul	95 674.	57 147.3	{3., 89 683.}	40.2687	1.12305
35	newly 3 weeks spec	179.	2426.84	{0., 456.}	1407.75	1.82935	newly 3 weeks cumul	229.	4601.92	{1., 600.}	2057.65	1.53133	newly 5 weeks spec	2514.	1400.86	{0., 1740.}	121.676	2.37998	newly 5 weeks cumul	3425.	7983.76	{1., 14 790.}	293.438	2.05353	newly 68 weeks cumul	95 744.	54 735.5	{1., 93 398.}	42.8315	1.51441
36	newly 3 weeks spec	451.	80.26	{0., 154.}	88.6918	1.63405	newly 3 weeks cumul	672.	134.88	{3., 267.}	87.3274	1.34282	newly 5 weeks spec	3163.	368.01	{0., 641.}	91.0013	2.15306	newly 5 weeks cumul	5077.	675.52	{4., 1233.}	90.2395	1.78465	newly 64 weeks cumul	99 501.	55 915.3	{5., 91 875.}	43.8043	1.199
37	newly 3 weeks spec	142.	303.59	{0., 168.}	264.866	1.2997	newly 3 weeks cumul	189.	448.82	{1., 281.}	278.349	1.08501	newly 5 weeks spec	2178.	908.24	{0., 520.}	117.712	2.05077	newly 5 weeks cumul	2952.	1974.89	{3., 1084.}	138.103	1.73241	newly 65 weeks cumul	99 424.	55 480.4	{3., 90 845.}	44.1982	1.21952
38	newly 3 weeks spec	1570.	352.11	{1., 888.}	87.0732	1.60067	newly 3 weeks cumul	2591.	604.89	{3., 1466.}	86.4566	1.46715	newly 5 weeks spec	6266.	1191.47	{0., 3411.}	85.3126	1.88881	newly 5 weeks cumul	12 039.	2473.7	{6., 6600.}	85.9656	1.67519	newly 62 weeks cumul	93 543.	60 910.2	{13., 91 757.}	34.8954	0.728331
39	newly 3 weeks spec	955.	115.84	{1., 232.}	92.7958	1.84061	newly 3 weeks cumul	1540.	191.08	{3., 409.}	92.1455	1.67544	newly 5 weeks spec	4980.	521.76	{1., 1188.}	93.0831	2.18601	newly 5 weeks cumul	8707.	972.12	{7., 2156.}	92.9179	1.94957	newly 64 weeks cumul	97 320.	63 647.	{1650., 90 905.}	34.6003	0.62407
40	newly 3 weeks spec	1152.	370.66	{0., 973.}	102.714	2.01189	newly 3 weeks cumul	1821.	533.54	{2., 1374.}	99.3904	1.80647	newly 5 weeks spec	6088.	1604.15	{0., 6802.}	97.4295	2.41319	newly 5 weeks cumul	10 814.	3035.04	{4., 10 904.}	98.8946	2.11287	newly 65 weeks cumul	99 384.	54 710.9	{4., 91 768.}	44.95	1.19109
41	newly 3 weeks spec	84.	2493.52	{0., 229.}	3005.4	1.58453	newly 3 weeks cumul	118.	4701.83	{0., 316.}	4017.62	1.36052	newly 5 weeks spec	1333.	1061.72	{0., 2213.}	133.443	2.14574	newly 5 weeks cumul	1781.	7214.54	{1., 9003.}	453.045	1.89019	newly 68 weeks cumul	96 330.	56 557.3	{1., 92 755.}	41.2879	1.48002
42	newly 3 weeks spec	2385.	2523.64	{962., 3612.}	34.1124	0.16683	newly 3 weeks cumul	4744.	4613.65	{1916., 6585.}	28.1427	0.149317	newly 5 weeks spec	6151.	6419.8	{2596., 8866.}	30.982	0.155668	newly 5 weeks cumul	14 873.	15 285.9	{6363., 21 532.}	31.0165	0.156996	newly 59 weeks cumul	96 441.	83 330.3	{42 231., 93 097.}	13.5945	0.0758198
43	newly 3 weeks spec	942.	262.71	{0., 462.}	100.218	1.85653	newly 3 weeks cumul	1522.	404.2	{2., 738.}	98.1853	1.66753	newly 5 weeks spec	4281.	1024.57	{0., 2249.}	96.06	2.15481	newly 5 weeks cumul	7799.	2002.7	{3., 3820.}	97.7405	1.8807	newly 64 weeks cumul	98 631.	59 133.1	{6., 89 789.}	40.0462	0.941814
44	newly 3 weeks spec	717.	202.87	{0., 240.}	103.614	1.89537	newly 3 weeks cumul	1097.	316.18	{2., 413.}	102.317	1.61983	newly 5 weeks spec	5449.	828.7	{0., 1308.}	96.1608	2.38251	newly 5 weeks cumul	8791.	1600.78	{3., 2287.}	97.9088	2.06324	newly 64 weeks cumul	99 299.	54 763.6	{4., 90 505.}	44.8498	1.1446
45	newly 3 weeks spec	514.	135.21	{0., 363.}	92.286	1.73707	newly 3 weeks cumul	734.	192.92	{1., 530.}	90.7221	1.53644	newly 5 weeks spec	4552.	1043.68	{0., 3826.}	93.6041	2.29748	newly 5 weeks cumul	6924.	1639.66	{1., 5559.}	93.5173	1.9762	newly 66 weeks cumul	99 357.	60 511.2	{2., 93 493.}	39.0972	1.23933
46	newly 3 weeks spec	247.	2186.32	{0., 779.}	940.883	1.86152	newly 3 weeks cumul	335.	2632.12	{1., 969.}	839.594	1.57032	newly 5 weeks spec	2717.	1892.94	{0., 6755.}	129.137	2.34831	newly 5 weeks cumul	3855.	7183.35	{1., 12 014.}	244.824	2.0715	newly 68 weeks cumul	99 095.	52 269.1	{1., 91 398.}	47.2536	1.63821
47	newly 3 weeks spec	977.	114.06	{1., 279.}	90.9806	1.68821	newly 3 weeks cumul	1597.	197.95	{3., 441.}	89.9706	1.52279	newly 5 weeks spec	4864.	450.03	{0., 1035.}	92.3838	2.03895	newly 5 weeks cumul	8755.	878.14	{5., 1987.}	91.8572	1.80658	newly 63 weeks cumul	96 045.	59 567.6	{7., 90 893.}	37.9795	0.945387
48	newly 3 weeks spec	364.	193.5	{0., 269.}	121.863	1.82166	newly 3 weeks cumul	527.	263.69	{1., 369.}	116.871	1.58494	newly 5 weeks spec	3183.	1128.62	{0., 2372.}	108.985	2.33852	newly 5 weeks cumul	4879.	1963.76	{2., 3578.}	112.779	2.02659	newly 66 weeks cumul	97 052.	59 228.8	{2., 92 033.}	38.9721	1.09492
49	newly 3 weeks spec	1900.	788.2	{0., 3336.}	91.3411	1.78556	newly 3 weeks cumul	3162.	1190.2	{3., 4923.}	88.0917	1.59811	newly 5 weeks spec	7611.	3120.57	{0., 13 630.}	87.8735	1.94239	newly 5 weeks cumul	14 764.	6102.2	{4., 26 566.}	89.244	1.76651	newly 64 weeks cumul	95 259.	61 160.1	{4., 91 590.}	35.796	0.899872
50	newly 3 weeks spec	943.	324.79	{0., 590.}	105.405	1.97819	newly 3 weeks cumul	1533.	465.61	{2., 994.}	101.818	1.75663	newly 5 weeks spec	5024.	1371.56	{0., 3180.}	98.6389	2.30368	newly 5 weeks cumul	8966.	2619.16	{3., 5295.}	100.442	2.04839	newly 65 weeks cumul	98 082.	59 347.3	{4., 91 199.}	39.4922	1.07879
